# Social isolation in patients with chronic limb-threatening ischemia: a cross-sectional study

**DOI:** 10.1038/s41598-023-29197-5

**Published:** 2023-02-02

**Authors:** Mitsuyoshi Takahara, Osamu Iida, Norihiko Ohura, Yoshimitsu Soga, Terutoshi Yamaoka, Nobuyoshi Azuma

**Affiliations:** 1 Department of Diabetes Care Medicine, Osaka University Graduate School of Medicine, 2-2 Yamadaoka, Suita, Osaka 565-0871 Japan; 2 Cardiovascular Center, Kansai Rosai Hospital, 3-1-69 Inabaso, Amagasaki, Hyogo 660-8511 Japan; 3 Department of Plastic, Reconstructive Surgery, Kyorin University School of Medicine, 6-20-2 Shinkawa, Mitaka, Tokyo 181-0004 Japan; 4 Department of Cardiology, Kokura Memorial Hospital, 3-2-1 Asano, Kokurakita-Ku, Kitakyushu, Fukuoka 802-0001 Japan; 5 Department of Vascular Surgery, Matsuyama Red Cross Hospital, 1 Bunkyo-Cho, Matsuyama, Ehime 790-8524 Japan; 6 Department of Vascular Surgery, Asahikawa Medical University, 2-1 Midorigaoka-Higashi, Asahikawa, 078-8510 Japan

**Keywords:** Cardiology, Diseases, Health care, Health occupations

## Abstract

Assistance by family members or friends plays important roles in the course of treating patients with chronic limb-threatening ischemia (CLTI), both during hospitalization and after discharge. The aim of this study was to reveal the prevalence of social isolation and to explore relevant clinical backgrounds in patients with CLTI presenting with tissue loss and requiring revascularization. We analyzed 413 patients registered in a multicenter study in whom revascularization were scheduled for CLTI with tissue loss. Social isolation was analyzed by assessing the residence status of the patients and the involvement of a trusted family member or friend in their daily lives and during hospitalization. Patients living alone accounted for 24.5% (95% confidence interval [CI] 20.1–28.8%) of the study population. Patients receiving welfare were more likely to live alone (P < 0.001). For patients living alone, 21.8% (95% CI 12.8–30.8%) met a trusted family member or friend in their daily lives less than once per year. Younger age and receiving welfare were independently associated with lower frequency of meeting the trusted person in their daily lives (both P < 0.05). The adjusted odds ratio of age and receiving welfare was 0.44 (95% CI 0.29–0.67) per 10-year increase and 3.47 (95% CI 1.43–8.44), respectively. During hospitalization, 9.9% (95% CI 6.8–13.0%) of the patients had no hospital visits by a trusted family member or friend on three key occasions: the patient’s first hospital visit, the preoperative explanation regarding the planned operation, and the day of the operation. Younger age and receiving welfare were independently associated with lower frequency of hospital visits by a family member or friend (both P < 0.05). The adjusted odds ratio of age and receiving welfare for no visit versus ≥ 1 visit was 0.51 (0.36–0.74) per 10-year increase and 5.29 (2.46–11.4), respectively. In conclusion, social isolation is common among patients with CLTI, especially younger patients and those on welfare. Practical countermeasures against social isolation are warranted in the management of CLTI.

## Introduction

Chronic limb-threatening ischemia (CLTI), especially that presenting with ischemic tissue loss, is the most advanced form of peripheral artery disease, and its prognosis is extremely poor^1–3^. Once CLTI develops, revascularization, either surgical or endovascular, is positioned as the first-line strategy for limb salvage^1–3^. However, tissue loss will not be cured immediately by revascularization; it takes a considerable time, usually weeks to months for tissue loss to completely heal after revascularization. In the course of treating patients with chronic wounds, both during hospitalization and after discharge, family support will play important roles^4^, as does interdisciplinary care^5^. Unfortunately, some patients with CLTI presenting with tissue loss tend to be socially isolated and get little family support. However, there is no available data on extent of social isolation in this population.

The aim of this study was to reveal the prevalence of social isolation and to explore relevant clinical backgrounds in patients with CLTI presenting with tissue loss and requiring revascularization.

## Methods

### Study population

This study was conducted using the database of the Wound-directed Angiosome RevasculaRIzation apprOach to patients with cRitical limb iSchemia (WARRIORS) study. The WARRIORS study is an ongoing prospective multicenter observational study of patients with CLTI due to atherosclerotic arterial disease, presenting with ischemic tissue loss with a skin perfusion pressure (SPP) < 40 mmHg, scheduled for infrapopliteal revascularization (either surgical or endovascular) at 29 centers in Japan. The study participants were registered at referral to the participating centers between October 2017 and June 2020.

This study was performed in accordance with the tenets of the Declaration of Helsinki and was approved by the ethics committee of Kansai Rosai Hospital, the principal research institution (approval date, September 26, 2017; approval number, #17C034g), and all the other centers where patients were registered. Informed consent was obtained from the participants or, if not possible, from their families. Of 450 registered patients, 37 patients staying in nursing homes were excluded. The remaining 413 patients were included in this study.

### Definitions

For the WARRIORS study, data relevant to social isolation, including the patients’ residence status and the involvement of a trusted family member or friend, were prospectively collected. The data were obtained from medical records or responses to interviews held during the registration. A trusted family member or friend was defined as a person who had the right and responsibility to share relevant patient information and was given the authority to participate in medical decisions or to give or withhold proxy consent, especially in an emergency in clinical settings. Residence status denotes whether a patient lived alone or with housemates. The involvement of a patient’s trusted family member or friend was evaluated from the perspective of two situations: their daily life and during hospitalization. Involvement in the daily life of a patient living alone was measured as the frequency of visits by a trusted family member or friend in their daily life. The frequency was categorized as follows: (1) everyday; (2) less frequent than everyday but at least once a week (one to 6 days a week); (3) less than once a week but at least once a month (once per week to once a month); (4) less than once a month but at least once a year (once per month to once a year); and (5) less than once per year. The categorization was prespecified in our registry, and the relevant data were accordingly collected; we provided the five categories as a predefined list of answer options to choose from, and collected the relevant data in a closed-ended manner. The categories were provided without further detailed definitions. The involvement of a patient’s trusted family member or friend during hospitalization was measured as the frequency of hospital visits by the person on three key medical occasions: (1) the patient’s first visit to the vascular center (at the referral); (2) the preoperative explanation regarding the planned operation to obtain relevant informed consent; and (3) the day of the operation. The frequency was categorized as all the time, twice, once, or never.

Patients with a non-ambulatory status were defined as patients who were not self-ambulatory, i.e., were in a wheelchair or were bed-ridden before the onset of CLTI. Patients with visual impairment were defined as patients who have difficulty performing visual foot inspections themselves due to impaired vision. Smoking status was determined based on whether a patient was a smoker at the onset of CLTI. Receiving welfare was defined as receiving public assistance in accordance with the relevant domestic law. Body mass index was calculated as weight in kilograms divided by height in meters squared. Dyslipidemia was defined as meeting one or more of the following criteria: (1) receiving anti-hyperlipidemic treatment; (2) low-density lipoprotein cholesterol level ≥ 140 mg/dl; (3) high-density lipoprotein cholesterol level < 40 mg/dl; and (4) triglyceride level ≥ 150 mg/dl^6^. Hypertension was defined as meeting one or more of the following criteria: (1) receiving anti-hypertensive treatment; (2) systolic blood pressure ≥ 140 mmHg; and (3) diastolic blood pressure ≥ 90 mmHg^7^. Diabetes mellitus was defined as meeting one or more of the following criteria: (1) receiving anti-diabetic treatment; (2) fasting plasma glucose level ≥ 126 mg/dl; (3) random plasma glucose level ≥ 200 mg/dl; and (4) glycated hemoglobin level ≥ 6.5%^8^. Dialysis dependence, i.e., end-stage renal disease that requires dialysis, included both dependence on hemodialysis and peritoneal dialysis. Coronary artery disease was defined as a history of myocardial infarction, symptomatic myocardial ischemia, or coronary revascularization. Chronic heart failure was defined as a history of hospitalization for the disease, current symptomatic heart failure, or treatment for the disease.

The severity of CLIT was evaluated according to the Wound, Ischemia, and foot Infection (WIfI) classification system^9^. The WIfI wound (W) and foot infection (fI) grades were determined by an independent plastic surgeon using photographs of pedal wounds and medical records to determine the presence of systemic inflammatory response syndrome. The WIfI ischemia (I) grade was determined based on the SPP. An SPP of 30–39 mmHg and < 30 mmHg was considered WIfI I-2 and I-3, respectively. As aforementioned, all study participants presented with ischemic tissue loss and an SPP < 40 mmHg, therefore being categorized as ≥ WIfI Wound grade 1 and ≥ Ischemia grade 2. We included one limb per patient in the analysis. For patients with bilateral CLTI, the limb with the more severe CLTI, which was judged by the WIfI class, was included in the analysis. The pressure sensation of the foot was assessed using a Semmes–Weinstein 5.07 monofilament at the following four podalic sites: the distal great toe and the first, third, and fifth metatarsal heads^10^. Loss of pressure sensation was defined as inability to correctly feel the pressure applied by the monofilament at any of the sites.

### Statistical analysis

Data are presented as medians and interquartile ranges for continuous variables or as frequencies and percentages for discrete variables, if not otherwise mentioned. A *P* value < 0.05 was considered statistically significant and 95% confidence intervals (CIs) are reported where appropriate. Of the 413 study participants, 3 (0.7%) had missing data regarding the frequency of hospital visits by a trusted family member or friend, whereas none had missing data on residence status and the frequency of meeting a trusted family member or friend in their daily lives. Missing data were addressed using multiple imputation with the chained equations method. During the procedure, we generated five imputed datasets and combined the analysis results according to Rubin’s rule.

The association between clinical characteristics and living alone was investigated using a simple logistic regression model. We also checked the effect size, which was calculated as *φ* of the chi-square test (equal to the square root of *χ*^2^/*n*) for dichotomous variables and *r* of Mann–Whitney's *U* test (equal to *z*-score divided by the square root of *n*) for continuous variables and ordered discrete variables. Note that both statistics of the effect size correspond to the correlation coefficient; the association was considered clinically significant when the statistic was larger than 0.2.

The association between clinical characteristics and lower frequency of meeting a trusted family member or friend in the daily lives of patients living alone was explored using the ordinal regression analysis. Note that the proportional odds assumption was not significantly violated for all variables (all P > 0.05 by the Brant test). The statistically significant variables in the simple regression model were subsequently entered in the multivariable regression model to examine their independent associations. We also checked the association by calculating Spearman’s correlation coefficients. The association was considered clinically meaningful when the absolute value of the correlation coefficient was larger than 0.2.

The ordinal regression analysis was also performed for the association between clinical characteristics and lower frequency of hospital visits by a trusted family member or friend on the three aforementioned key occasions. However, the Brant test confirmed that the proportional odds assumption was significantly violated for some clinical characteristics (P = 0.008 for receiving welfare, P = 0.007 for hemoglobin levels, and P > 0.05 for the other variables). The crude association was alternatively evaluated using Spearman’s correlation coefficients. The statistically significant variables in the crude analysis were subsequently entered in the separate multivariable logistic regression models for the dichotomized outcome measure (i.e., ≤ 2 visits versus 3 visits, ≤ 1 visit versus ≥ 2 visits, and no visit versus ≥ 1 visit). All statistical analyses were performed using R version 4.1.1 (R Development Core Team, Vienna, Austria).

### Ethics approval and consent to participate

The current study, involving humans, was performed in accordance with the tenets of the Declaration of Helsinki. The study was approved by the ethics committee of Kansai Rosai Hospital (approval number, #17C034g). Informed consent was obtained from the participants. Patients who were unable to provide informed consent by themselves due to their dementia were not excluded from the study, but were also eligible to participate in the study. Those patients were enrolled in the study, if informed consent was obtained from their families.

## Results

**Figure 1:**
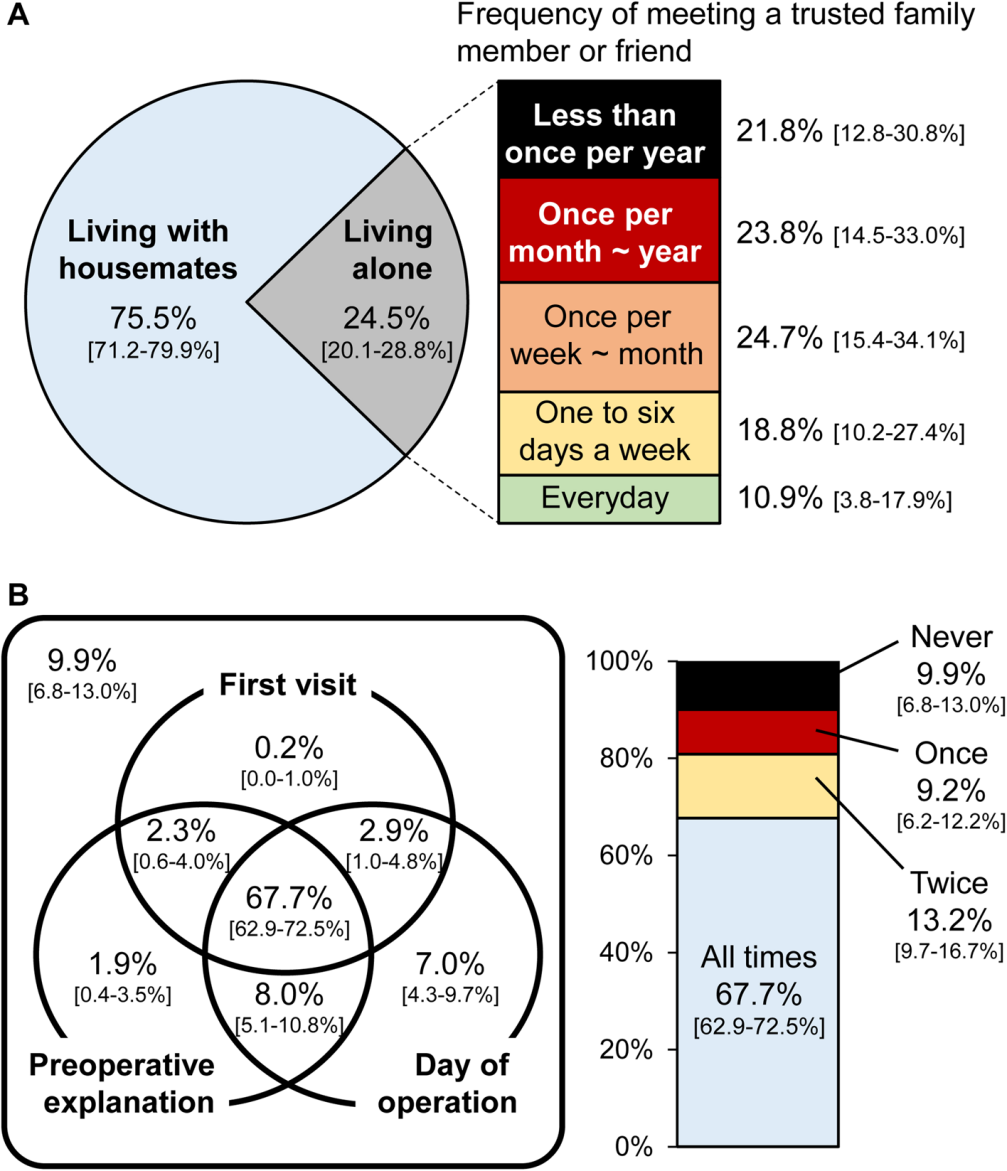
Social isolation of the study population (*n* = 413). (**A**) Shows the residence status of the study population (the pie chart on the left) and the frequency of patients living alone meeting a trusted family member or friend (the band graph on the right). (**B**) Shows the frequency of hospital visitation of patients living alone by a trusted family member or friends on three key occasions: (1) the patient’s first visit to the vascular center; (2) the preoperative explanation regarding the planned operation; and (3) the day of the operation (the Venn diagram on the left and the band graph on the right). Data are proportions [95% confidence intervals].

The clinical characteristics of the study population are summarized in Table 1. The median age of the study population was 74 (interquartile range 68–80) years old and 64.1% of them were male. The prevalence of diabetes mellitus and renal failure on dialysis was 74.1% and 60.5%, respectively. WIfI clinical stages 2, 3, and 4 were recorded in 5.0%, 29.1%, and 65.9% of the study population, respectively. Figure 1A shows the residence status and the frequency of meeting a trusted family member or friend in a patient’s daily life. Patients living alone accounted for 24.5% (95% CI 20.1–28.8%) of the overall population. Those living alone did not frequently meet a trusted family member or friend in their daily lives; 21.8% (12.8–30.8%) met the person less than once per year. As illustrated in Fig. 1B, 9.9% (6.8–13.0%), 9.2% (6.2–12.2%), 13.2% (9.7–16.7%), and 67.7% (62.9–72.5%) of the study population were visited by a trusted family member or friend in the hospital on none, one, two, and all three key occasions, respectively.

**Table 1:** Characteristics of the study population (*n* = 413).

Age (years)	74 (68–80)
Male	273 (66.1%)
Non-ambulatory	77 (18.6%)
Visual impairment	52 (12.6%)
Receiving welfare	46 (11.1%)
Smoking	36 (8.7%)
Body mass index (kg/m^2^)	21.5 (19.2–24.6)
Dyslipidemia	253 (61.3%)
Hypertension	351 (85.0%)
Diabetes mellitus	306 (74.1%)
Renal failure on dialysis	250 (60.5%)
Coronary artery disease	189 (45.8%)
Stroke	84 (20.3%)
Chronic heart failure	82 (19.9%)
Hemoglobin (g/dL)	11.2 (9.9–12.4)
Albumin (g/dL)	3.4 (3.0–3.8)
Contralateral major amputation	22 (5.3%)
Bilateral CLTI	40 (9.7%)
Revascularization strategy	
Bypass surgery	134 (32.4%)
Endovascular therapy	279 (67.6%)
WIfI classification: wound	
W-1	167 (41.5%)
W-2	183 (45.5%)
W-3	52 (12.9%)
WIfI classification: ischemia	
I-2	93 (22.5%)
I-3	320 (77.5%)
WIfI classification: foot infection	
fI-0	69 (17.2%)
fI-1	121 (30.1%)
fI-2	195 (48.5%)
fI-3	17 (4.2%)
WIfI classification: clinical stage	
Stage 2	20 (5.0%)
Stage 3	117 (29.1%)
Stage 4	265 (65.9%)
Loss of pressure sensation	129 (36.1%)

Data are median (interquartile range), or number (percentage). Thirteen patients (3.1%) were missing data on albumin level, 11 (3.7%) were missing data on WIfI classification except ischemia grade, and there were missing data on pressure sensation for 56 (13.6%) limbs. *CLTI* chronic limb-threatening ischemia, *WIfI* wound, ischemia, and foot infection.

**Figure 2:**
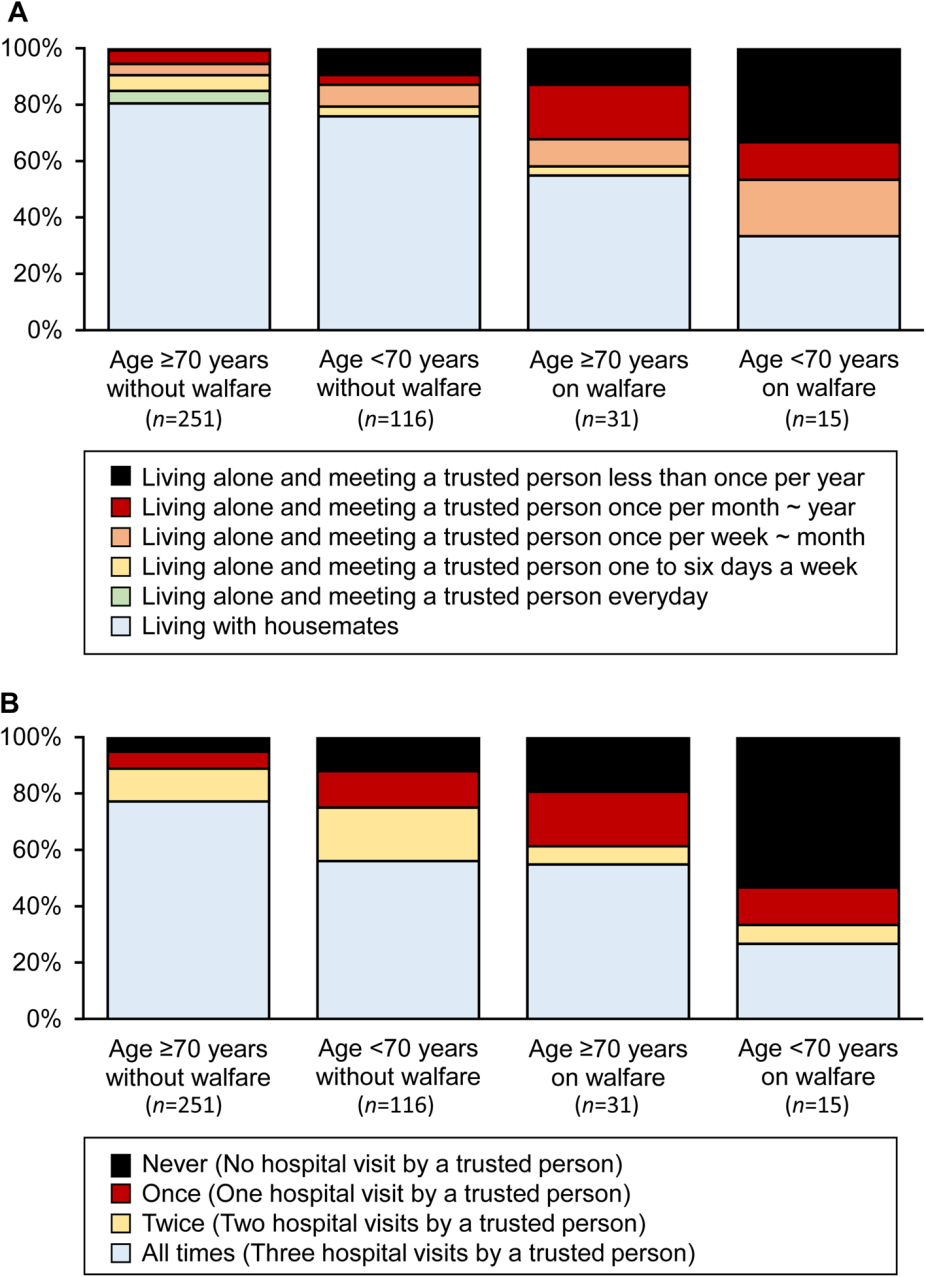
Social isolation according to age and receipt of welfare in the study population (*n* = 413). (**A**) Shows the proportion of patients living with housemates and those living alone (segmentized according to the frequency of meeting a trusted family member or friend). (**B**) Shows the frequency of the hospital visitation of patients living alone by a trusted family member or friends on three key occasions: (1) the patient’s first visit to the vascular center; (2) the preoperative explanation regarding the planned operation; and (3) the day of the operation.

Table 2 demonstrates the association between clinical characteristics and living alone. Receiving welfare was the only variable significantly associated with living alone (P < 0.001) (odds ratio 4.11; 95% CI 2.19–7.72). Receiving welfare had an effect size of larger than 0.2, whereas the other variables had an effect size of smaller than 0.1. For patients living alone, age, sex, receiving welfare, and smoking had a significant crude association with lower frequency of meeting a trusted family member or friend (all P < 0.05), and at the same time had an absolute value of Spearman’s correlation coefficient of larger than 0.2 (Table 3). The multivariable analysis showed that younger age and receiving welfare were independently associated with lower frequency of meeting a trusted family member or friend (P < 0.001 and P = 0.006) (Table 4). The adjusted odds ratio of age and receiving welfare was 0.44 (0.29–0.67) per 10-year increase and 3.47 (1.43–8.44), respectively. Table 5 shows the crude association between clinical characteristics and lower frequency of hospital visits by a trusted family member or friend. Age and receiving welfare had a significant crude association with the frequency (both P < 0.05), and at the same time had an absolute value of Spearman’s correlation coefficient of larger than 0.2. The multivariable analysis showed that younger age and receiving welfare were independently associated with the frequency (Table 6). The adjusted odds ratios of age (per 10-year increase) and receiving welfare were 0.54 (0.42–0.69; P < 0.001) and 2.68 (1.41–5.10; P = 0.003) for ≤ 2 visits versus 3 visits, 0.49 (0.37–0.66; P < 0.001) and 4.93 (2.52–9.66; P < 0.001) for ≤ 1 visit versus ≥ 2 visits, and 0.51 (0.36–0.74; P < 0.001) and 5.29 (2.46–11.4; P < 0.001) for no visit versus ≥ 1 visit, respectively. Patients who live alone and rarely meet a trusted family member or friend in their daily lives, and those who were rarely visited in the hospital by a trusted family member or friend, were commonly younger and on welfare (Fig. 2).

**Table 2:** Crude association between clinical characteristics and living alone in the overall study population (*n* = 413).

	Unadjusted odds ratio	Effect size (*φ* or *r*)
Age (per 10 years)	0.94 [0.74–1.20] (P = 0.63)	0.02
Male	0.76 [0.48–1.21] (P = 0.25)	0.05
Non-ambulatory	1.10 [0.63–1.95] (P = 0.73)	0.01
Visual impairment	1.30 [0.68–2.48] (P = 0.43)	0.03
Receiving welfare	4.11 [2.19–7.72] (P < 0.001)	0.22
Smoking	1.62 [0.78–3.37] (P = 0.20)	0.05
Body mass index (per 5 kg/m^2^)	0.93 [0.70–1.23] (P = 0.62)	0.03
Dyslipidemia	1.01 [0.64–1.60] (P = 0.98)	0.00
Hypertension	0.63 [0.35–1.13] (P = 0.12)	0.07
Diabetes mellitus	0.88 [0.53–1.46] (P = 0.63)	0.02
Renal failure on dialysis	0.94 [0.59–1.48] (P = 0.79)	0.01
Coronary artery disease	1.16 [0.74–1.81] (P = 0.52)	0.03
Stroke	0.61 [0.33–1.13] (P = 0.12)	0.07
Chronic heart failure	1.00 [0.57–1.75] (P = 0.99)	0.00
Hemoglobin (per 1 g/dL)	1.03 [0.92–1.16] (P = 0.61)	0.02
Albumin (per 1 g/dL)	1.11 [0.75–1.64] (P = 0.60)	0.03
Contralateral major amputation	1.17 [0.44–3.07] (P = 0.75)	0.00
Bilateral CLTI	0.89 [0.41–1.93] (P = 0.76)	0.01
Endovascular (versus surgical)	1.19 [0.73–1.93] (P = 0.49)	0.03
WIfI: wound	0.96 [0.68–1.36] (P = 0.83)	0.01
WIfI: ischemia	1.15 [0.67–1.99] (P = 0.61)	0.02
WIfI: foot infection	0.89 [0.67–1.18] (P = 0.42)	0.04
WIfI: clinical stage	0.87 [0.59–1.28] (P = 0.49)	0.02
Loss of pressure sensation	0.98 [0.59–1.62] (P = 0.92)	0.01

Odds ratios, derived from the logistic regression model, are presented together with 95% confidence intervals. *CLTI* chronic limb-threatening ischemia, *WIfI* wound, ischemia, and foot infection.

**Table 3:** Crude association of clinical characteristics with lower frequency of meeting with a trusted family member or friend in a subgroup who lived alone (*n* = 101).

	Spearman’s correlation coefficient *ρ*	Simple ordinal regression model	
		Unadjusted odds ratio	Brant test
Age (per 10 years)	− 0.44 (P < 0.001)	0.39 [0.26–0.58] (P < 0.001)	P = 0.14
Male	0.30 (P = 0.003)	3.05 [1.46–6.38] (P = 0.003)	P = 0.32
Non-ambulatory	0.02 (P = 0.85)	1.09 [0.44–2.70] (P = 0.84)	P = 0.46
Visual impairment	− 0.02 (P = 0.86)	0.92 [0.35–2.41] (P = 0.86)	P = 0.35
Receiving welfare	0.33 (P = 0.001)	4.05 [1.75–9.36] (P = 0.001)	P = 0.57
Smoking	0.21 (P = 0.036)	3.59 [1.12–11.5] (P = 0.031)	P = 0.76
Body mass index (per 5 kg/m^2^)	0.15 (P = 0.13)	1.33 [0.90–1.99] (P = 0.16)	P = 0.85
Dyslipidemia	− 0.03 (P = 0.80)	0.91 [0.45–1.85] (P = 0.79)	P = 0.88
Hypertension	− 0.05 (P = 0.59)	0.78 [0.32–1.88] (P = 0.58)	P = 0.67
Diabetes mellitus	0.17 (P = 0.085)	2.01 [0.91–4.40] (P = 0.083)	P = 0.32
Renal failure on dialysis	− 0.02 (P = 0.87)	0.94 [0.46–1.91] (P = 0.87)	P = 0.061
Coronary artery disease	0.09 (P = 0.39)	1.36 [0.68–2.73] (P = 0.38)	P = 0.69
Stroke	− 0.11 (P = 0.26)	0.58 [0.23–1.51] (P = 0.27)	P = 0.67
Chronic heart failure	0.07 (P = 0.47)	1.43 [0.57–3.55] (P = 0.44)	P = 0.42
Hemoglobin (per 1 g/dL)	0.05 (P = 0.65)	1.08 [0.89–1.29] (P = 0.44)	P = 0.37
Albumin (per 1 g/dL)	− 0.07 (P = 0.52)	0.81 [0.45–1.46] (P = 0.48)	P = 0.20
Contralateral major amputation	0.04 (P = 0.73)	1.24 [0.33–4.63] (P = 0.75)	P = 1.00
Bilateral CLTI	− 0.17 (P = 0.085)	0.37 [0.11–1.20] (P = 0.096)	P = 0.93
Endovascular (versus surgical)	− 0.01 (P = 0.95)	0.97 [0.45–2.10] (P = 0.95)	P = 0.60
WIfI: wound	0.17 (P = 0.10)	1.64 [0.95–2.86] (P = 0.078)	P = 0.39
WIfI: ischemia	− 0.19 (P = 0.061)	0.45 [0.19–1.06] (P = 0.068)	P = 0.30
WIfI: foot infection	0.04 (P = 0.72)	1.08 [0.70–1.66] (P = 0.73)	P = 0.37
WIfI: clinical stage	0.13 (P = 0.21)	1.30 [0.75–2.23] (P = 0.35)	P = 0.33
Loss of pressure sensation	0.00 (P = 1.00)	1.00 [0.47–2.12] (P = 1.00)	P = 0.26

Odds ratios, derived from the ordinal regression analysis, are presented together with 95% confidence intervals. *CLTI* chronic limb-threatening ischemia, *WIfI* wound, ischemia, and foot infection.

**Table 4:** Adjusted association of clinical characteristics with lower frequency of meeting with a trusted family member or friend in a subgroup who lived alone (*n* = 101).

	Adjusted odds ratio
Age (per 10 years)	0.44 [0.29–0.67] (P < 0.001)
Male	1.57 [0.70–3.49] (P = 0.27)
Receiving welfare	3.47 [1.43–8.44] (P = 0.006)
Smoking	2.31 [0.70–7.62] (P = 0.17)

Data are adjusted odds ratios [95% confidence intervals] (P values), derived from the multivariable ordinal regression analysis. The multivariable ordinal regression model included all the variables in the table, which had a statistically significant crude association, and simultaneously had an absolute value of Spearman’s correlation coefficient of larger than 0.2 (see Table 3).

**Table 5:** Crude association of clinical characteristics with lower frequency of hospital visitation by a trusted family member or friend in the overall study population (*n* = 413).

	Spearman’s correlation coefficient *ρ*	Simple ordinal regression model	
		Unadjusted odds ratio	Brant test
Age (per 10 years)	− 0.27 (P < 0.001)	0.52 [0.41–0.66] (P < 0.001)	P = 0.82
Male	0.05 (P = 0.27)	1.28 [0.83–1.97] (P = 0.27)	P = 0.81
Non-ambulatory	− 0.02 (P = 0.70)	0.90 [0.54–1.52] (P = 0.70)	P = 0.31
Visual impairment	− 0.03 (P = 0.51)	0.81 [0.43–1.52] (P = 0.52)	P = 0.93
Receiving welfare	0.21 (P < 0.001)	3.83 [2.10–7.00] (P < 0.001)	P = 0.008
Smoking	0.08 (P = 0.12)	1.75 [0.88–3.45] (P = 0.11)	P = 0.48
Body mass index (per 5 kg/m^2^)	0.07 (P = 0.18)	1.21 [0.95–1.55] (P = 0.12)	P = 0.14
Dyslipidemia	0.04 (P = 0.40)	1.20 [0.79–1.82] (P = 0.40)	P = 0.89
Hypertension	− -0.05 (P = 0.29)	0.75 [0.44–1.29] (P = 0.30)	P = 0.69
Diabetes mellitus	− 0.04 (P = 0.42)	0.83 [0.52–1.30] (P = 0.41)	P = 0.22
Renal failure on dialysis	0.09 (P = 0.069)	1.48 [0.97–2.26] (P = 0.069)	P = 0.98
Coronary artery disease	0.03 (P = 0.48)	1.16 [0.77–1.73] (P = 0.48)	P = 0.79
Stroke	− 0.09 (P = 0.075)	0.63 [0.37–1.06] (P = 0.082)	P = 0.15
Chronic heart failure	− 0.05 (P = 0.29)	0.75 [0.44–1.28] (P = 0.29)	P = 0.96
Hemoglobin (per 1 g/dL)	− 0.09 (P = 0.059)	0.92 [0.82–1.02] (P = 0.12)	P = 0.007
Albumin (per 1 g/dL)	0.02 (P = 0.64)	1.02 [0.72–1.44] (P = 0.93)	P = 0.64
Contralateral major amputation	0.07 (P = 0.13)	1.91 [0.83–4.37] (P = 0.13)	P = 0.92
Bilateral CLTI	− 0.03 (P = 0.61)	0.83 [0.40–1.70] (P = 0.60)	P = 0.46
Endovascular (versus surgical)	0.07 (P = 0.16)	1.38 [0.88–2.15] (P = 0.16)	P = 0.78
WIfI: wound	0.01 (P = 0.89)	1.03 [0.75–1.41] (P = 0.88)	P = 0.60
WIfI: ischemia	− 0.01 (P = 0.90)	0.97 [0.60–1.58] (P = 0.90)	P = 0.50
WIfI: foot infection	0.03 (P = 0.53)	1.08 [0.83–1.42] (P = 0.55)	P = 0.75
WIfI: clinical stage	0.02 (P = 0.62)	1.06 [0.74–1.51] (P = 0.75)	P = 0.45
Loss of pressure sensation	− 0.05 (P = 0.39)	0.81 [0.50–1.31] (P = 0.39)	P = 0.87

Odds ratios, derived from the ordinal regression analysis, are presented together with 95% confidence intervals. *CLTI* chronic limb-threatening ischemia, *WIfI* wound, ischemia, and foot infection.

**Table 6:** Adjusted association of clinical characteristics with lower frequency of hospital visitation by a trusted family member or friend in the overall study population (*n* = 413).

	Adjusted odds ratio for ≤ 2 visits versus 3 visits	Adjusted odds ratio for ≤ 1 visit versus ≥ 2 visits	Adjusted odds ratio for No visit versus ≥ 1 visit
Age (per 10 years)	0.54 [0.42–0.69] (P < 0.001)	0.49 [0.37–0.66] (P < 0.001)	0.51 [0.36–0.74] (P < 0.001)
Receiving welfare	2.68 [1.41–5.10] (P = 0.003)	4.93 [2.52–9.66] (P < 0.001)	5.29 [2.46–11.4] (P < 0.001)

Data are adjusted odds ratios [95% confidence intervals] (P values), derived from the multivariable logistic regression analysis. The multivariable logistic regression model included all the variables in the table, which had a statistically significant crude association, and simultaneously had an absolute value of Spearman’s correlation coefficient of larger than 0.2 (see Table 4).

## Discussion

The present study revealed social isolation in patients with CLTI presenting with ischemic tissue loss and requiring revascularization. The results showed that patients living alone accounted for 24.5% (20.1–28.8%) of the overall population. We also found that patients receiving welfare were more likely to live alone. Among the patients living alone, 21.8% (12.8–30.8%) met a trusted family member or friend in their daily lives less than once per year. Younger age and receiving welfare were independently associated with lower frequency of meeting a trusted family member or friend in the daily lives of the patients. Regarding hospitalization, 9.9% (6.8–13.0%) of the overall population were not visited in the hospital by a trusted family member or friend. Younger age and receiving welfare were again independently associated with lower frequency of hospital visits by a trusted family member or friend. Our findings indicate that a considerable number of patients with CLTI are socially isolated, both in daily life and during hospitalization. To the best of our knowledge, this is the first report on social isolation in patients with CLTI.

The present study revealed that social isolation is common in patients with CLTI. Although social isolation as a concept was established decades ago and has recently started attracting increasing attention, there is no standard, international, widely used, or cross-culturally valid measure of the concept^13^. The definition of social isolation therefore varied from study to study, which made it difficult to compare the prevalence among studies. Exceptionally, living alone is a marker for social isolation that has been widely adopted. In Japan, the prevalence of living alone was reported to be 9–18% in community-dwelling older adults^11–14^ and 15–21% in patients with cardiovascular diseases^15–19^. The corresponding prevalence in the study population was 24.5% (95% CI 20.1–28.8%), suggesting that the prevalence was at least not lower (or possibly slightly higher) than in other populations. Social isolation is a common issue faced in the clinical management of CLTI.

The present study also showed that receiving welfare was an associated factor, indicating an association between low socioeconomic status and social isolation. Although the causal relationship between these factors remained unproven, our finding is consistent with previous reports^20^. Individuals with low socioeconomic status are at a disadvantage in shaping their living conditions and physical environments, which provide access and opportunities to develop and maintain social connections^20^.

Age was also an associated factor. Age was inversely associated with both lower frequency of meeting a trusted family member or friend in the daily life of a patient living alone and lower frequency of hospital visitation by a trusted family member or friend, indicating that younger patients with CLTI were more likely to be socially isolated than older patients. The reason why age was inversely associated with social isolation remained unknown. The present finding is contrary to some previous studies showing that social isolation increases with age^20,21^. The positive association between age and social isolation in these previous studies was observed in a general population, and was reasonably attributed to the fact that life transitions and disruptive life events (such as retirement; bereavement of a spouse, partner or friends; migration of children; and disability or loss of mobility) are more likely to affect older people^20–24^. In contrast, the present study analyzed a population with CLTI. Social isolation is a risk factor for various diseases including peripheral artery disease^25,26^, indicating that socially isolated patients will likely develop the disease earlier (i.e., at younger age). Although the association between social isolation and CLTI remained unrevealed, socially isolated patients might develop CLTI similarly earlier. When one focuses on a population with CLTI, patients in whom social isolation contributed to their CLTI development might be more commonly seen in a younger subgroup. That might be why the prevalence of social isolation was inversely associated with age in this population. Indeed, some studies reported a similarly inverse association between social isolation and age in a population with a disease for which social isolation is known as a risk factor^27,28^. The finding that the association in a diseased population was opposite to that observed in a general population might be warranted, although the true mechanism remained unknown.

There was no significant association between social isolation and visual impairment, loss of pressure sensation, or non-ambulatory status in the present study. This indicates that vulnerable patients, who are expected to require ample support from others for foot care and daily living, are as commonly socially isolated as non-vulnerable patients. Furthermore, diabetes mellitus and other cardiovascular risk factors were not associated with social isolation; in other words, these comorbidities were as prevalent in socially isolated patients as in patients with healthy social relationships. Social isolation increases the risk of poorer diet, lower physical activity, and poor adherence to medical treatments^22^, which can considerably interfere with the control of those comorbidities in the CLTI population. As recommended in clinical guidelines, patients with CLTI require appropriate control of diabetes mellitus and other cardiovascular risk factors, as well as healthy diet, exercise, and preventive foot care^2^. Social isolation is a clinical issue that cannot be ignored in the management of patients with CLTI.

One major limitation of the present study was that the association between social isolation and clinical outcomes was not analyzed. Currently, data on clinical outcomes are not available in this registry. Recent studies have demonstrated that social isolation has negative effects on health, prognosis, and well-being in various populations^22,29–35^, plausibly due to poor diet, low physical activity, poor adherence to medical treatments, and reduced psychological stress-buffering effect of social support^22^. It has also been reported that social isolation increases resource utilization and costs^36^. Future studies are needed to reveal the association between social isolation and clinical outcomes in this population.

The present study has several other limitations. First, although social isolation as a concept was established decades ago and has recently started attracting increasing attention, there is no standard, international, widely used, or cross-culturally valid measure of the concept^22^. Social isolation denotes the objective state of having a reduced network of kin and non-kin relationships and thus, few or infrequent interactions with others^22^. We tentatively measured social isolation using residence status and the involvement of a trusted family member or friend in a patient’s life. However, these measurements are not yet externally validated. Furthermore, we categorized the frequency of visits by a trusted family member or friend into five categories, not according to any evidence or established classification system, but simply on our empirical basis. The categorization was prespecified in our registry, and the relevant data were accordingly collected. We were therefore unable to analyze the relevant data according to a different categorization or definition. Second, our database did not include a general population or a population with other atherosclerotic diseases. We were therefore unable to compare the prevalence of social isolation between the CLTI population and other populations. Third, the data on treatment before the index referral, including primary foot care and patient education, were unavailable in this study. In addition, the reason for living alone or the reason for lower frequency of daily contact and hospital visits by a trusted family member or friend of a patient are unknown. Fourth, we included patients with CLTI scheduled for infrapopliteal revascularization. Although most patients with CLTI undergo infrapopliteal revascularization in clinical settings^37–39^, whether the findings of this study would be applicable for patients undergoing revascularization of other vascular territories is unknown. Furthermore, this study was conducted in Japan. Other countries, with different healthcare systems and societal structures, would have different trends in social isolation. Thus, future studies conducted in other countries are warranted.

## Conclusions

The present study provides relevant information regarding social isolation in patients with CLTI presenting with ischemic tissue loss and requiring infrapopliteal revascularization in Japan. Socially isolation was common in patients with CLTI, especially in younger patients and those on welfare. Considering the high prevalence of social isolation in the CLTI population, practical countermeasures against social isolation are necessary in the management of CLTI.

## Data Availability

The datasets generated and/or analyzed during the current study are not publicly available due to ethical reasons but are available from the corresponding author on reasonable request and with permission of the ethics committee of the participating institutions.

## Abbreviations

CI: Confidence interval
CLTI: Chronic limb-threatening ischemia
SPP: Skin perfusion pressure
WARRIORS: Wound-directed angiosome revascularization approach to patients with critical limb ischemia
WIfI: Wound, ischemia, and foot infection
